# Understanding the relationships between teacher mindfulness, work engagement, and classroom emotions

**DOI:** 10.3389/fpsyg.2022.993857

**Published:** 2022-09-29

**Authors:** Wei Tao

**Affiliations:** School of English Studies, Zhejiang International Studies University, Hangzhou, China

**Keywords:** teacher mindfulness, work engagement, classroom emotion, positive emotion, negative emotion

## Abstract

This cross-sectional study investigated the relationships between teacher mindfulness, work engagement, and classroom emotions composed of positive and negative emotions. A sample of 498 Chinese primary, secondary, and high school teachers completed an anonymous online questionnaire. Descriptive analysis, bivariate correlation, and a series of regression equations were conducted to analyze the data. The results indicate that teacher mindfulness, work engagement, and classroom emotions are all at the intermediate level, and significantly correlated. The effect of teacher mindfulness on classroom emotions is partially mediated by work engagement. In addition, negative emotions partially mediate the effect of teacher mindfulness on work engagement, while positive emotions fully mediate it. These results highlight the importance of fostering teacher mindfulness through mindfulness-based intervention, developing teacher emotion regulation competence in teacher education, and cultivating supportive classroom and school culture for teachers to experience more positive emotions from students and administrators.

## Introduction

Today, education is not only supposed to develop students’ skills for future work and life but is also required to promote their wellbeing. Teacher wellbeing greatly impacts student academic performance and wellbeing (Wellbeing Australia, 2011). Thus, promoting teacher wellbeing has become a pursuit of teacher education and an issue in teacher development research. Previous studies take a problem-based approach in examining teacher wellbeing, focusing mainly on the negative sides of teacher life such as burnout, anxiety, and retention (Paterson and Grantham, 2016; Liu et al., 2022). Such a prevalent deficit approach results from the negative psychology perspective that aims at detecting and dealing with psychological problems. Although detecting and dealing with psychological problems could indirectly lead to teacher wellbeing, researchers are recently proposing that a strength approach might better foster teacher wellbeing (Roffey, 2012).

The positive psychology perspective provides such an approach. Positive Psychology is “a science of positive, subjective experience, positive individual traits. And, positive institutions promise to improve the quality of life and prevent the pathologies that arise when life is barren and meaningless” (Seligman and Csikszentmihalyi, 2000, p. 5). The basic assumption of the positive psychology perspective is that “human goodness and excellence are as authentic and common as are disease, disorder, and distress” (Hoy and Tarter, 2011, p. 428). Under the guidance of this assumption, studies from the positive psychology perspective inquire into the nature of effectively functioning individuals or organizations (Sheldon and King, 2001).

Based on principles of the positive psychology perspective, research on both positive and negative aspects of teacher traits and emotions can better attain the goal of promoting teacher wellbeing. The past few years witnessed many studies guided by these principles (Chu et al., 2021; Liu et al., 2021). But further studies are needed since many positive aspects of teacher traits and emotions in specific contexts such as China are under-examined. With the purpose of enriching studies from the positive psychology perspective and facilitating teacher wellbeing, this paper studies the relationships between teacher mindfulness and teacher emotions in the Chinese context.

## Literature review

### Teacher mindfulness

Mindfulness is a human quality that affects wellbeing (Keng et al., 2011). It refers to “the awareness that emerges through paying attention on purpose, in the present moment and nonjudgmentally to the unfolding of experience moment by moment” (Kabat-Zinn, 2003, p. 145). As a significant positive factor, mindfulness in general could be measured by many scales that were developed, with which as tools; relationship between mindfulness and emotions was found (Singh et al., 2016).

Mindfulness has been sporadically researched in the teacher development field for about 20 years. However, it was not until TIME’s declaration of the mindful revolution in 2014 that teacher mindfulness research started to burgeon. Previous studies mainly analyzed the relationships between mindfulness and positive and negative emotions. Some studies concluded that teacher mindfulness and mindfulness-based interventions were negatively related to negative emotions, such as stress, burnout, depression, and anxiety (Flook et al., 2013; Crain et al., 2017; Klingbeil and Renshaw, 2018; Schussler et al., 2018). In contrast, another group of studies concluded that teacher mindfulness and mindfulness-based interventions were positively related to positive emotions such as resilience and empathy (Meiklejohn et al., 2012; Rechtschaffen, 2014; Lomas et al., 2017).

The studies revealed the close relationships between teacher mindfulness (mindfulness-based interventions included) and teacher emotions. But there are two limitations to these studies. Firstly, the interactive feature of teacher mindfulness has not been considered since these studies’ mindfulness scales measure only intrapersonal mindfulness. The newly developed mindfulness in teaching scale, which contains both teacher intrapersonal and interpersonal mindfulness (Frank et al., 2016; Kim and Singh, 2018) can bridge the gap. The studies did not fully analyze the relationship between teacher mindfulness and positive emotions.

### Teacher emotions

Teaching is an emotional practice since emotion and teaching are inextricably linked to one another (Hargreaves, 1998; Zembylas, 2004; Schutz and Zembylas, 2009). Teacher emotions refer to feelings teachers experience in their work. Regarding the interaction of intrapersonal mindfulness and interpersonal mindfulness, this paper investigates the two main categories of teacher emotions that might interact with one another, work engagement and classroom emotions, the former being more intrapersonal and the latter being more interpersonal.

#### Work engagement

Work engagement lies in “the positive pole of worker’s wellbeing” (Schaufeli et al., 2002, p. 71). It refers to “a positive, fulfilling, work-related state of mind that is characterized by vigor, dedication, and absorption” (Schaufeli et al., 2002, p. 74). To be specific, vigor refers to high levels of energy and mental resilience while working, the willingness to invest effort in one’s work, and persistence even in the face of difficulties. Dedication refers to a sense of significance, enthusiasm, inspiration, pride, and challenge; absorption refers to the state of being fully concentrated on and deeply engrossed in one’s work, whereby time passes quickly and one has difficulties with detaching oneself from work (Schaufeli et al., 2002). As the opposite concept of burnout (Maslach and Leiter, 1997), work engagement developed due to the result of the positive psychology turn.

In teacher development, work engagement has been less researched than teacher burnout. Previous studies mainly focused on structural components of work engagement and reciprocal relationships between work engagement and other factors. Following Schaufeli et al. (2002), several studies have been conducted to explore the structural features of work engagement in different groups of participants (Perera et al., 2018; Tomás et al., 2018). Many studies also inquired into the reciprocal relationship between work engagement and other positive and negative emotions (Rothmann, and Hamukang’andu, L., 2013; Eldor and Shoshani, 2016; Burić and Macuka, 2018).

The conclusions drawn in these studies provided implications as follows. Firstly, the Utrecht work engagement scale has been confirmed by studies in different contexts and thus can be applied to studies on teacher work engagement. Secondly, work engagement has been found to be an emotional factor closely related to other emotional factors. However, there is a shortage of studies on the relationship between work engagement and teacher mindfulness.

#### Classroom emotions

Classroom emotions refer to teachers’ and students’ perceptions of emotional experiences while teaching and learning inside the classroom and interacting with one another (Titsworth et al., 2010). As classrooms witness the major part of the interaction between teachers and students and others in school, classroom emotions greatly affect the wellbeing of teachers and students.

At the time of this study, most studies on classroom emotions mainly targeted students (Titsworth et al., 2010; Dewaele and Dewaele, 2020; Li et al., 2020). Very few studies have investigated teacher classroom emotions. A few studies analyzed the links between teacher emotions and students’ emotions and learning outcomes (Hagenauer et al., 2015; Frenzel et al., 2021), and the relationships between teacher classroom emotions and their own job satisfaction, self-efficacy, and work engagement (Hu et al., 2016).

The studies examined positive and negative classroom emotions experienced by teachers and highlighted the interconnection between teacher classroom emotions and their work and student learning. However, there is a remarkable lack of studies on the relationship between teacher classroom emotions and teacher mindfulness.

To sum up, the past years have witnessed an increase of studies on teacher mindfulness and teacher emotions (work engagement and classroom emotions in particular). But the relationship between the two in the Chinese context has been under researched.

The Chinese context is worth studying for at least two reasons. First, emotional experiences and emotion regulation are culture-sensitive (Gross, 2007; Yin, 2016). Asian cultures (Chinese culture included) emphasize individuals’ harmonious interdependence with others, but American culture emphasizes individuals maintaining independence by attending to the self-and discovering and expressing their unique inner attributes (Markus and Kitayama, 1991; Hosotani and Imai-Matsumura, 2011). The cultural differences affect teacher emotional experiences and emotion regulation and the relationship between teacher mindfulness and teacher emotions. Thus, ample evidence is needed before the wide promotion of teacher development by fostering mindfulness in the Chinese context. Second, as a country with the largest number of students in the whole world, teachers in China experience numerous emotions because of the national curriculum reform (Lee and Yin, 2011), the heart-consuming feature of teaching and the influence of the social norms (Yin and Lee, 2012), the classroom and collegial interaction, and the educational policies, changes, and imbalance of teachers’ lives (Chen, 2016), and many other factors. Research about teacher emotion and its regulation in the Chinese context is growing (Chan and Hui, 1995; Chan, 2006; Jiang et al., 2016; Yin, 2016), However, there is limited research regarding on the relationship between teacher mindfulness and teacher emotional experiences and emotion regulation (the few examples are Siu et al., 2014; Hue and Lau, 2015). Thus, it is crucial to investigate the relationship between teacher mindfulness and teacher emotions in the Chinese context.

### Aims and research questions

The current study investigates the relationship between teacher mindfulness and teacher emotions (work engagement and classroom emotions in particular) in the Chinese context. Unlike previous studies that focused only on the intrapersonal dimension of teacher mindfulness, this study investigates both intrapersonal and interpersonal mindfulness (Frank et al., 2016; Kim and Singh, 2018). Correspondingly, teacher emotion in this study reflects work engagement and classroom emotions composed of positive and negative emotions that might interact with one another, with work engagement being more intrapersonal and classroom emotions being more interpersonal.

To be specific, this paper was guided by three sets of research questions.

1. What is the level of teacher mindfulness, work engagement, and classroom emotions? Are the three variables significantly correlated?
2. Does teacher mindfulness have a significant effect on classroom emotions (including positive and negative emotions)? If yes, is it mediated by work engagement?
3. Does teacher mindfulness have a significant effect on work engagement? If yes, is it mediated by positive and negative emotions?

## Materials and methods

### Participants

Around 526 primary, secondary, and high school teachers in mainland China participated in this study, of which 498 provided valid data. As there is obvious difference between university teachers and primary, secondary, and high school teachers in China, this paper does not collect data from university teachers. Participant demographics are summarized in Table 1. Among the 498 participants, there are 402 female and 96 male teachers. 320 teachers have been teaching for no more than 10 years, 118 have 11–20 years of teaching experience, and only 60 have been teaching for more than 21 years. About 90 teachers are from primary schools, 142 are from middle schools, and 266 are from high schools. 133 teachers work in the countryside, while 365 work in schools in the city. 35, 255, and 208 teachers self-assess to have weak, average, or strong teaching competence. Though the sample is not in perfect proportion to the teacher population in China in terms of demographics, it can depict an overall picture of the relationship between teacher mindfulness and teacher emotions in this and similar contexts.

**Table 1:** Participant demographics.

Variable	*N*	%
Gender		
Female	402	80.72
Male	96	19.28
Years of teaching		
≤10	320	64.26
11–20	118	23.70
≥21	60	12.06
School level		
Primary school	90	18.07
Middle school	142	28.51
High school	266	53.41
School location		
Countryside	133	26.71
City	365	73.29
Self-assessment of teaching competence		
Weak	35	7.03
Average	255	51.20
Strong	208	41.76

*n* = 498.

### Measures

An online questionnaire with items to collect data on demographics, teacher mindfulness, work engagement, and classroom emotions was administered to the participants. The demographics focused on gender, years of teaching, school level, school location, and self-assessment of teaching competence.

#### Teacher mindfulness

In previous studies, teacher mindfulness was depicted by adapted versions of the widely used definition of mindfulness in general. Correspondingly, it was measured by scales that were used for general mindfulness, which focused only on the intrapersonal dimension. This paper adopted the 14 items scale covering both intrapersonal and interpersonal dimensions, which was specified for teachers in America (Frank et al., 2016) and confirmed in both Chinese and Korean contexts (Kim and Singh, 2018; Ding et al., 2020), although the Chinese version added two more items.

This paper applied the Chinese version of the 14 items. The first nine items related to present-centered awareness involving elements of awareness, attentiveness and focus on the present moment (Frank et al., 2016), reflecting teacher intrapersonal mindfulness. The rest five items represent an open, accepting, and receptive disposition and approach to student-teacher interactions (Frank et al., 2016), reflecting teacher interpersonal mindfulness. The integration of the two dimensions can help measure teacher mindfulness comprehensively. The 14 items were rated on a five-point Likert scale from 1 (never true) to 5 (always true). The current sample displayed good internal reliability with a Cronbach’s alpha of 0.800.

#### Work engagement

Schaufeli et al. (2002) developed a scale of 17 items to measure work engagement in various occupational contexts (Schaufeli et al., 2002). This study used the adapted Chinese version of the scale with 15 items for teachers (Zhang and Gan, 2005). Among the 15 items, six items measure vigor, four items measure dedication, and five items measure absorption. The three dimensions have been found to be highly correlated (Zhang and Gan, 2005). Thus, this paper computes all the 15 items as a whole. In this study, the 15 items were rated on a five-point Likert scale from 1 to 5, different from the original seven-point Likert scale. The overall internal reliability for the current sample was excellent, with a Cronbach’s alpha of 0.943.

#### Classroom emotions

To measure teacher positive and negative emotions in class, this paper follows the classic way of measuring positive and negative emotions separately (Watson and Clark, 1988), and specifically follows Hu et al. (2016) in the Chinese context. Eight items (three for positive emotions and five for negative emotions) were designed to draw data on emotions of joy, love, satisfaction, anger, anxiety, nervousness, frustration, and helplessness (Hu et al., 2016). The eight items were rated on a five-point Likert scale from 1 (never) to 5 (always). High scores represent strong emotions. The sample displayed good internal reliability, with a Cronbach’s alpha of 0.902 for positive emotions and 0.846 for negative emotions.

### Procedure

The study adopts the convenience sampling strategy. Once the questionnaire was set online, an introduction was written before the items, explaining the nature of the study, the anonymous feature of the questionnaire, and the process to do the questionnaire. Then it was sent to teachers by the authors directly or through teacher educators who have close contact with the teachers. Teachers read the introduction and decided to participate or not. Those who were willing to participate completed the questionnaire within 10–15 min.

After deleting invalid data, the questionnaires were sorted and input into SPSS (Version 16). Then negatively worded items were reverse-scored and tested the internal reliability. Mean scores for the variables were computed. After these preparations, descriptive analysis, bivariate correlation, and a series of regression equations (Baron and Kenny, 1986) were conducted to analyze the data.

## Results

### Level of teacher mindfulness, work engagement, and classroom emotions and the correlation between the three variables

To answer the first set of research questions, this paper conducted descriptive analysis and bivariate correlation. The results are displayed in Table 2.

**Table 2:** Description and correlation.

Variable	TM	WE	PE	NE
TM	-	0.427^**^	0.487^**^	−0.319^**^
WE		-	0.780^**^	−0.263^**^
PE			-	−0.250^**^
NE				-
*M*	3.5469	3.0922	3.2771	2.8924
*SD*	0.4563	0.7756	0.8038	0.6601

*n* = 498. TM refers to teacher mindfulness; WE refers to work engagement; PE refers to positive emotions; NE refers to negative emotions.

** *p* < 0.01.

In general, teacher mindfulness, work engagement, and classroom emotions are all at the intermediate level. To be specific, the mean score for teacher mindfulness is 3.5469 (SD = 0.4563). It is not very high, indicating that the participating teachers do not have strong mindfulness and there is big room for further development. The mean score for work engagement is 3.0922 (SD = 0.7756). It is fairly low, revealing that the teachers have weak work engagement and much space for improvement. The mean score for positive emotions is 3.2771 (SD = 0.8038), indicating that teachers have not experienced enough positive emotions in class. The mean score for negative emotions is 2.8924 (SD = 0.6601), revealing that the teachers have experienced quite a few negative emotions in class.

As is shown in Table 2, teacher mindfulness, work engagement and classroom emotions are significantly correlated. Teacher mindfulness significantly correlates with work engagement (*r* = 0.427, *p* < 0.01) and positive emotions (*r* = 0.487, *p* < 0.01) at the intermediate level. It is also significantly correlated with negative emotions (*r* = −0.319, *p* < 0.01) at the weak level. Work engagement is strongly correlated with positive emotions (*r* = 0.780, *p* < 0.01), and weakly correlated with negative emotions (*r* = −0.263, *p* < 0.01). Positive and negative emotions are significantly correlated (*r* = −0.250, *p* < 0.01).

### The effect of teacher mindfulness on classroom emotions and the mediation of work engagement

**Figure 1:**
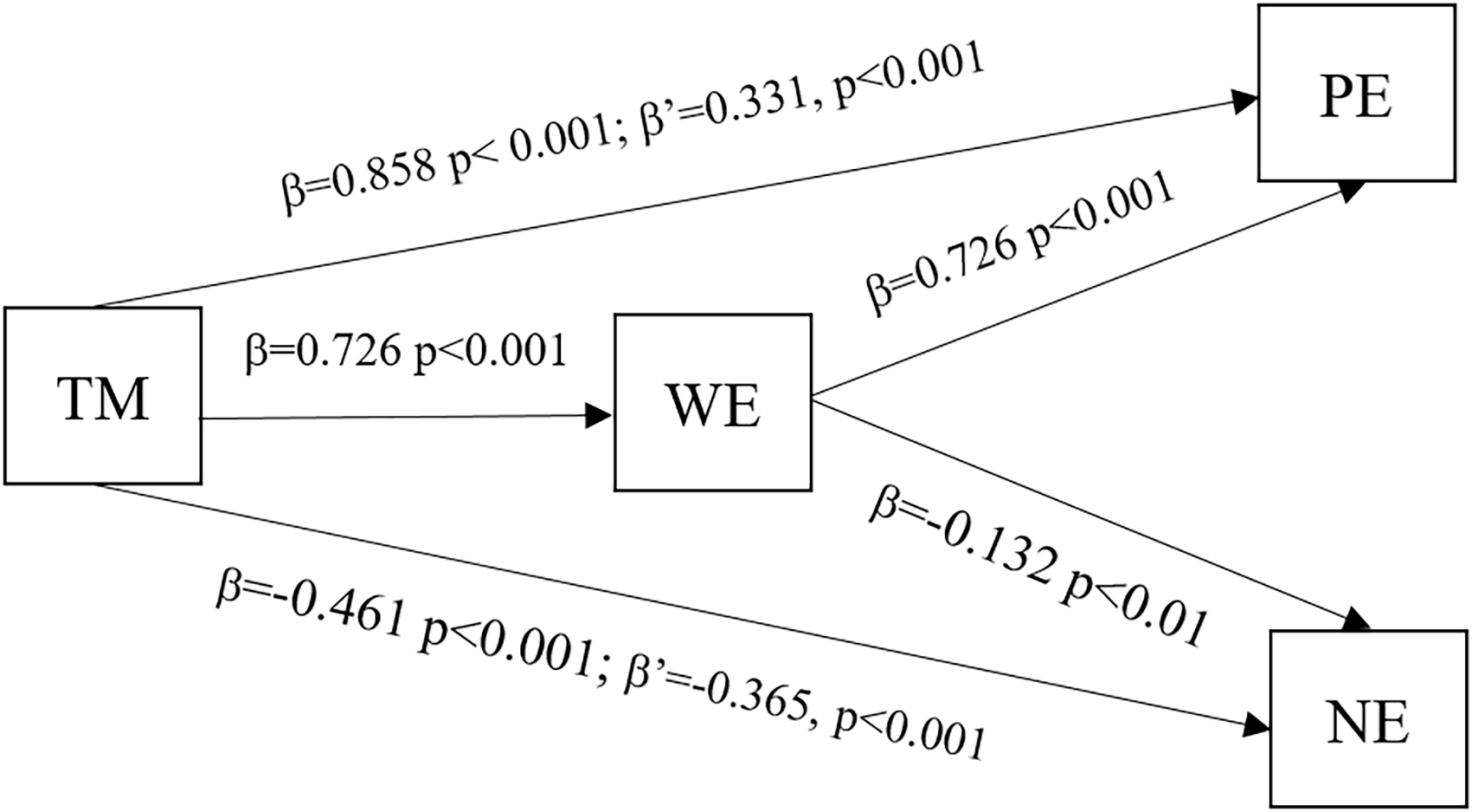
The effect of teacher mindfulness on classroom emotions and the mediation of work engagement.

In order to answer the second set of research questions, this paper conducted a series of regression equations. The results are presented in Figure 1. It is shown that teacher mindfulness has strong and intermediate effects on positive and negative emotions, and the effects are both partially mediated by work engagement.

To report in detail, teacher mindfulness has a strong total effect on positive emotions, which can be reflected in the equation: PE = 0.858TM + 0.235. Teacher mindfulness also has a strong effect on work engagement: WE = 0.726TM + 0.516. When teacher mindfulness and work engagement are taken together, the effect of teacher mindfulness on positive emotions is still significant but weakened: PE = 0.331TM + 0.726WE−0.140. That is to say, work engagement partially mediates the effect of teacher mindfulness on positive emotions.

Besides, teacher mindfulness has intermediate total effect on negative emotions: NE = −0.461TM + 4.529. When it goes together with work engagement, its effect is still significant but weakened in intensity, as shown in the equation: NE = −0.365TM−0.132WE+4.597. Thus, work engagement also partially mediates the effect of teacher mindfulness on negative emotions.

### The effect of teacher mindfulness on work engagement and the mediation of classroom emotions

**Figure 2:**
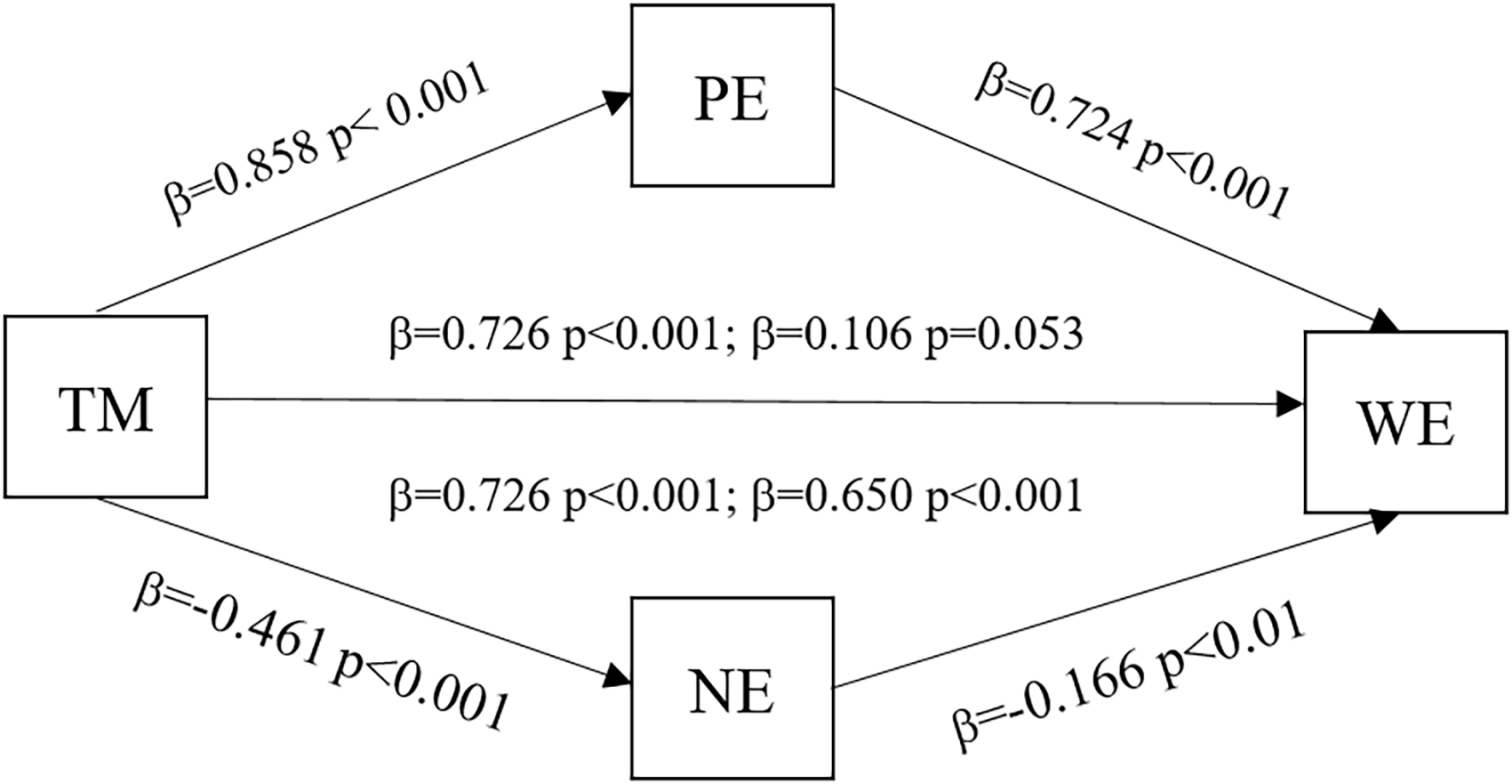
The effect of teacher mindfulness on work engagement and the mediation of classroom emotions.

Answers to the third set of research questions are also drawn from a series of regression equations. As can be seen in Figure 2, teacher mindfulness’ effects on work engagement, positive and negative emotions are confirmed. Besides, the effects of teacher mindfulness on work engagement are mediated by both positive and negative emotions.

As the data show, when teacher mindfulness and positive emotions are combined, the effect of teacher mindfulness on work engagement drastically reduces to be insignificant (*p* = 0.053 > 0.05). Thus, positive emotions fully mediate the effect of teacher mindfulness on work engagement.

When teacher mindfulness and negative emotions are integrated, the effect of teacher mindfulness on work engagement is weakened but still significant: WE = 0.650TM−0.166NE+1.268. It reveals the partial mediation of negative emotions on teacher mindfulness’ effect on work engagement.

## Discussion

**Figure 3:**
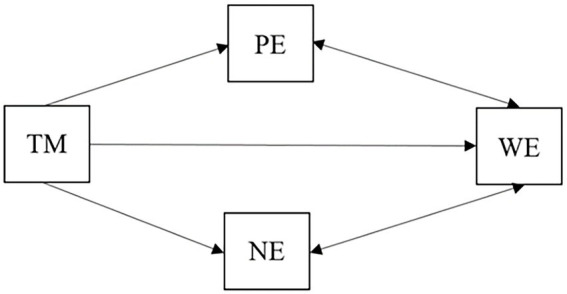
A summary of the results of this study.

The major results of this study are 3-fold. First, teacher mindfulness, work engagement, and classroom emotions are at the intermediate level and significantly correlated. Second, the effect of teacher mindfulness on classroom emotions is mediated by work engagement. Third, the effect of teacher mindfulness on work engagement is mediated by positive and negative emotions. They can be summarized in Figure 3, and they corroborate with the results in previous studies but also reveal unique features.

The level of teacher mindfulness is intermediate (with a mean score of 3.5469). It is similar to the results of the two studies that developed and validated the mindfulness in teaching scale in America and South Korea since the 14 items got average scores between 3.30 and 4.19 in Frank et al. (2016) and average scores between 3.40 and 4.24 in Kim and Singh (2018). It echoes teacher mindfulness measured by the five-factor mindfulness questionnaire in Crain et al. (2017), which got mean scores between 3.13 and 3.17 at three times for the control group and mean scores between 3.30 and 3.65 for the experimental group that experienced mindfulness-based intervention. It also corroborates teacher mindfulness measured by a scale integrating the five-factor mindfulness questionnaire and the five items of teacher interpersonal mindfulness in Jennings et al. (2017), which got a mean score of 3.55 before intervention, and mean scores of 3.56 and 3.68 for the control and experimental groups after the intervention. The results of this study and those relevant studies reveal that there is room and the possibility for the development of teacher mindfulness. A possible reason for the present level of teacher mindfulness is that they have not got any training on their mindfulness during their pre-service and in-service teacher education. Besides, they themselves have seldom done any intentional practice on their mindfulness, and most teachers do not know a viable way to do it.

Teacher work engagement reported by the participants is fairly weak. The mean score of 3.0922 is a bit lower than 4.45 drawn from the seven-point Likert scale in Hu et al. (2016) and much lower than the total score of 55.03 drawn from the 16 items five-point Likert scale in Li et al. (2015). The large proportion of teachers with fewer years of teaching experience in this study accounts for the relative weakness. As work engagement emerged to be the opposite concept of burnout (Maslach and Leiter, 1997), the low level of teacher work engagement indirectly confirms the high level of teacher burnout in China (Zhang and Yu, 2007), reflecting the view that teacher stress and burnout have become a crisis in education (Farber, 1991). In a word, teacher work engagement is in need of improvement. A possible reason is that about 58% of participants self-assess to have weak and average teaching competence. The lack of strong teaching competence affects teacher vigor, dedication, and absorption.

Both positive emotions and negative emotions in this study are intermediate. The mean score for positive emotions is lower than that in Hu et al. (2016), while the mean score for negative emotions is higher than that in it (the means scores in that study are correspondingly 3.61 and 2.43). Again, the large proportion of teachers with fewer years of teaching experience explains such a gap. Both positive emotions and negative emotions in this study are weaker than those in Chen (2016), which adopted six-point Likert scale to study teacher emotions within and without the class (it got a mean score of 5.36 for joy, 4.28 for love, 4.63 for sadness, 4.55 for anger, and 4.31 for fear). That is to say, compared with the micro-context of the classroom, teachers might experience more complex emotions in the meso-context of the school. All in all, there is space to promote positive emotions and reduce negative emotions for teachers in this study. There are two reasons. One is the fact that teaching is an emotional practice. It is normal for teachers to experience both positive and negative emotions. The other is that teachers are not good at emotion regulation, and have seldom been trained to do it.

The correlations and mediations reveal both direct and indirect effects of teacher mindfulness on teacher emotions. The direct effects of teacher mindfulness on work engagement, positive emotions and negative emotions confirm the findings that mindfulness and mindfulness-based intervention can reduce negative teacher emotions such as stress, burnout, depression, and anxiety (Flook et al., 2013; Hue and Lau, 2015; Crain et al., 2017; Klingbeil and Renshaw, 2018; Schussler et al., 2018). It also confirms that it promotes their positive emotions such as resilience, empathy, job satisfaction, and physical/psychological symptoms (Meiklejohn et al., 2012; Rechtschaffen, 2014; Siu et al., 2014; Lomas et al., 2017). It also verifies the original intention to develop mindfulness-based intervention: Offering an environment for participants to experience effective methods for facing, exploring, and relieving suffering, such as stress, pain, and illness (Kabat-Zinn, 2003). Such an effect has been supported by neurobiological evidence. Results of experiments showed that participants of mindfulness-based intervention experienced significant increases in left-sided anterior activation, which was a pattern associated with positive affect (Davidson et al., 2003). To sum up, fostering teacher mindfulness through mindfulness-based intervention is an effective practice to help teachers regulate their emotions.

The indirect effect of teacher mindfulness on work engagement through classroom emotions and on classroom emotions through work engagement, especially the full mediation effect of positive emotions, pinpoint the essential roles of teacher intrapersonal and interpersonal emotions in affecting one another’s generation and regulation. This partially echoes the finding that teacher classroom emotions mediate the effect of emotion regulation strategies on work engagement in Hu et al. (2016). The reciprocal mediation effect between teacher intrapersonal and interpersonal emotions can be indirectly explained by the reciprocal relationships between personal resources (self-efficacy, organization-based self-esteem, and optimism). It can also be justified by job resources (autonomy, social support, supervisory coaching, performance feedback, and opportunities for professional development), and work engagement found in employees in other fields (Xanthopoulou et al., 2009). According to the ecological dynamics system theory of teacher emotion, emotions generate in teacher dynamic appraisal of how successful personal goal pursuits are within a particular context (Cross and Hong, 2012; Schutz, 2014). It is a result of the interaction between personal and job resources. Such a standpoint also explains the reciprocal mediation effect between work engagement and positive and negative emotions. Thus, helping teachers to develop emotion regulation competence and creating a school context that is beneficial in reducing negative emotions and facilitating positive emotions are also important for teacher mindfulness to affect emotion regulation in a deeper manner.

## Conclusion

The present study aims to investigate the relationships between teacher mindfulness, work engagement, and classroom emotions that are composed of positive and negative emotions. The results indicate that teacher mindfulness, work engagement, and classroom emotions for this sample of Chinese teachers are at the intermediate level, and the three variables are significantly correlated. Work engagement partially mediates the effects of teacher mindfulness on positive and negative emotions. Reciprocally, positive emotions fully mediate the effect of teacher mindfulness on work engagement, and negative emotions partially mediate it.

The results provide implications for teacher professional development practice. Firstly, fostering teacher mindfulness through mindfulness-based intervention is a viable strategy to be implemented among teachers in China and similar contexts. It can directly and indirectly reduce negative emotions and promote positive emotions and work engagement. Secondly, developing teacher emotion regulation competence should be set as a goal of teacher education. Emotion regulation competence can help teachers control their emotions and deepen teacher mindfulness on work engagement and positive and negative emotions in class. Together with teaching competence, teacher mindfulness and a more substantial emotion regulation competence can make teacher personal resources more positive. Thirdly, students and school administrators are encouraged to cultivate a supportive classroom and school culture for teachers to experience more positive emotions. It can make teacher job resources more positive.

The present study contributes to the literature in three ways. Firstly, this is the very first Chinese sample to use the mindfulness in teaching scale in an empirical study (Frank et al., 2016; Kim and Singh, 2018). The good internal reliability supports the instrument to be applied to the measurement of teacher mindfulness in China and similar contexts. Secondly, these are the first few studies that examined the effect of teacher mindfulness on teacher emotions in the Chinese context. The findings provide preliminary evidence for the importance of fostering teacher mindfulness through mindfulness-based intervention. Thirdly, this study analyses the reciprocal mediation effects between teacher intrapersonal and interpersonal emotions. The reciprocal mediation effects emphasize the role of emotions themselves in emotion regulation.

The limitations of the study are 4-fold. The cross-sectional nature of it prevents causal analysis. Future studies can research the relationship over time by using longitudinal design. As mentioned, the sample is not in perfect proportion to the teacher population in China. Proportionately stratified sampling will help gain more representative results. The data is self-reported. An integration of it with qualitative data will make the analysis deeper. The discussion part compares mean scores in other studies and this study, but does not conduct meta-analysis. A meta-analysis of the scores in different studies will make the comparison more convincing.

## Data availability statement

The raw data supporting the conclusions of this article will be made available by the authors, without undue reservation.

## Ethics statement

The studies involving human participants were reviewed and approved by Zhejiang International Studies University. The patients/participants provided their written informed consent to participate in this study.

## Author contributions

WT conceived of the presented idea, and planned the study, collected and analyzed the data, and wrote and revised the article.

## Funding

This work was supported by Zhejiang Research Institute of Education Science-Project of Educational Science under Grant (number 2022SCG406) and the National Social Science Fund of China under Grant (number 21BYY120).

## Conflict of interest

The author declares that the research was conducted in the absence of any commercial or financial relationships that could be construed as a potential conflict of interest.

## Publisher’s note

All claims expressed in this article are solely those of the authors and do not necessarily represent those of their affiliated organizations, or those of the publisher, the editors and the reviewers. Any product that may be evaluated in this article, or claim that may be made by its manufacturer, is not guaranteed or endorsed by the publisher.
